# The Meaning Given to Bioethics as a Source of Support by Physicians Who Care for Children Who Require Long-Term Ventilation

**DOI:** 10.1177/10497323221083744

**Published:** 2022-03-29

**Authors:** Denise Alexander, Mary B. Quirke, Carmel Doyle, Katie Hill, Kate Masterson, Maria Brenner

**Affiliations:** 1 School of Nursing and Midwifery, The University of Dublin, 8809 Trinity College Dublin, Dublin, Ireland; 2 School of Nursing, Midwifery and Health Systems, 8797 University College Dublin, Dublin, Ireland

**Keywords:** ethics, moral perspectives, long-term health care, intensive care unit, assistive technology

## Abstract

The role and potential of bioethics input when a child requires the initiation of technology dependence to sustain life is relatively unknown. In particular, little is understood about the meaning physicians give to bioethics as a source of support during the care of children in pediatric intensive care who require long-term ventilation (LTV). We used a hermeneutic phenomenological approach to underpin the collection and analysis of data. Unstructured interviews of 40 physicians in four countries took place during 2020. We found that elements of trust, communication and acceptance informed the physicians’ perceptions of the relationship with bioethics. These ranged from satisfaction to disappointment with their input into critical decisions. Bioethics services have potential to help physicians gain clarity over distressing and complex care decisions, yet physicians perceive the service inconsistently as a means of support. This research provides a sound basis to guide more beneficial interactions between clinicians and bioethics services.

## Introduction

Over the last 20 years, an increasing number of children with serious conditions have survived in and beyond pediatric intensive care due to the introduction of life-sustaining technology (Amin et al., 2014; Cohen et al., 2011; Weiss et al., 2016). Many of these children rely on technology, such as long-term ventilation (LTV), to sustain their lives (Alexander, Quirke, et al., 2021; Fontana et al., 2013). Alongside survival rates, morbidity rates among these children has also increased. This may have important personal, family, and wider social consequences (Alexander, Quirke, et al., 2021). The process of initiating LTV in a child, whether this is invasively in the form of a tracheostomy, for example, or non-invasively through bilevel positive airway pressure (BiPAP) or continuous positive airway pressure (CPAP) ventilation, may involve the discussion of complex ethical issues around survival and quality of life. There are limited data around the influences on initiation of technology dependence and little is understood about the impact of personal values or opinions on decisions made (Novotny et al., 2014). Ethical principles such as respect for autonomy, beneficence, non-maleficence, and justice remain central to care delivery (Beauchamp & Childress, 1989; Gillon, 1994), as well as respect for shared decision-making with the family of the sick child (Johnson et al., 2015). However, making decisions around the initiation of life-sustaining technology can be particularly difficult involving factors beyond the clinical, such as values and beliefs, which may differ between clinician teams and the patient and family, as well as across and within countries (Carnevale, 2005; Shapiro et al., 2017; Thorne et al., 2018; Van Manen, 2014a). Decision-making for children requiring life-sustaining technology is becoming increasingly challenging in the context of the increase in availability and potential of life-sustaining technology for children (Alexander, Quirke, et al., 2021; Gold et al., 2011; Hajibabaee et al., 2016; Johnson et al., 2015).

One important means of navigating the challenges surrounding the decision whether to initiate technology dependence may be by the inclusion of bioethics services in the discussions (Johnson et al., 2015; Kesselheim et al., 2010; Morrison et al., 2015). The bioethics services aim to aid clinicians to make decisions by ensuring that current national and international ethical guidelines and frameworks are recognized and adhered to in discussions (Leland et al., 2020; Thomas et al., 2015); as well as to help mediate where there are differences in opinion between clinicians and the family of the child (Morrison et al., 2015). Access to bioethics services, however, is not consistent internationally, nor across different national hospital sites (McDougall & Notini, 2016; Morrison et al., 2015; Thomas et al., 2015). They may vary in their make-up depending on the institution with provisions such as a formal ethics committee, informal small group-discussions or one-to-one discussions with an individual bioethicist, or a health professional with an interest in, or qualification in, bioethics (Cottle et al., 2017; Kesselheim et al., 2010; Streuli et al., 2014; Thomas et al., 2015). In addition, in a time of rapid technological development and initiation, understanding the relationship between the clinicians and bioethics services is important, not least to guide the evolution of pertinent support for the physicians.

This study was part of a 5-year program of research that aims to understand the influences more fully on initiating life-sustaining technology in a child, in contrasting health, legal and socio-political systems. A major part of the program of research comprises a phenomenological approach towards the working lives of the clinicians caring for children at this point of care. In this study, we sought to interpret the meaning given to bioethics services from the perspective of physicians in pediatric intensive care units (PICUs) who care for children who require LTV to sustain life. This is important to improve understanding and utility between the two vital elements in the initiation of technology dependence.

## Methods

### Ethics

The research ethics committee of the project institution granted ethical approval for this study, and the local research ethics committees at each interview site also approved the research. The ethical permission from the project host institution protected any participants who contributed from outside of the interview sites. All participants received written information about the purpose of the study, and their right to withdraw at any time with no consequences to themselves. Participants provided oral and written or electronic informed consent to participate in the study.

### Theoretical Framework

The philosophical work of van Manen in phenomenological research informed our framework for investigation and analysis (Van Manen, 1997, 2014a, 2014b, 2017a). Using a hermeneutic phenomenological approach allowed us discern the meaning given to the care of children by physicians working in an environment where profound and life-changing decisions about children’s lives and health are made. This framework is particularly applicable to complex issues in child health research (Roscigno & Swanson, 2011; van Manen, 2014b, 2017b). The understanding of the interplay of activities within the context of a phenomenon, that this theoretical framework allows, offers the potential to understand the whole, rather than focusing on only part of the experience (Carel, 2011; Rodriguez & Smith, 2018). Van Manen’s approach facilitates a hermeneutic understanding of the day-to-day experiences of physicians caring for children who depend upon technology to sustain life (Van Manen, 1997). Six activities interplay to allow a deep understanding of the meaning of everyday existence. These are (1) turning to a phenomenon, making a commitment to understanding that world; (2) investigating experience as we live it rather than as we conceptualize it; (3) reflecting on the essential themes, which characterize the phenomenon; (4) describing the phenomenon through the art of writing and rewriting; (5) maintaining a strong and oriented relation to the phenomenon; and (6) balancing the research context by considering the parts and the whole (Van Manen, 1997 pp. 39–42). This technique allowed us to explore and interpret rich meaning from the data (Van Manen, 1997), while recognizing and recording any personal influences during the interpretation (Norlyk & Harder, 2010). The richness of meaning in the data allow what van Manen describes as the lived experience of individuals to be illuminated (Van Manen, 2014a). In order to access the lived experience of any participant to build a picture of the phenomenon of initiating technology dependence in children to sustain life, we aimed to understand each experience as it was relayed. As far as possible, we rejected any predetermined, theoretical or conceptual influences on any meaning we interpreted. We approached this goal by analyzing the stories of the participants through the different lenses of lived experiences, otherwise known as existentials: lived other, lived space, lived body, lived time, and lived things. These helped us to interpret the meaning of what was said and capture the essences underlying the phenomenon described (Van Manen, 2017a).

### Rigor

Guba and Lincoln’s (1982) four criteria of credibility, transferability, dependability and confirmability guided our research to ensure rigor in recruitment, data gathering, and interpretation of qualitative findings. We engaged with the research sites well in advance of the interview process. Local site gatekeepers were very familiar with the research aims and the inclusion criteria for interview and guided us in recruiting participants. We developed the interview protocol based on current literature (Alexander, Quirke, et al., 2021a; Alexander, Eustace-Cook, et al., 2021b). Participants were guided to recall their experience of initiating technology dependence to sustain a child’s life and the interviewer listened for engagement with bioethics services. During the interview, we allowed participants plenty of time to relate their experiences without interruption, although we prompted for more detail where necessary. After each interview, the researcher completed a reflective observation sheet. We continued to conduct interviews in each site until no new information emerged. Once complete, we anonymized each interview by job role and country. We recorded coding in NVIVO version 12, in phases guided by the project qualitative analysis framework. Bi-weekly meetings during data collection allowed us to discuss emerging findings and refine our focus in the interviews. These bi-weekly meetings continued during the interpretation and analysis of the data to discuss findings.

### Data Collection

#### Sampling criteria

All participants were physicians who had experience of caring for children in PICUs who are, or who are likely to become, technology dependent in order to sustain their life. Participants worked in, or very closely aligned to the PICU in the care of the children. In order to narrow down the focus of such a large inquiry, we used LTV as an exemplar for all technology dependence, as this means of technology dependence is one of the most commonly initiated in order to sustain life (Amin et al., 2014; Wallis et al., 2011).

#### Recruitment

We identified international sites in four countries for inclusion in the study: Australia, Ireland, the Netherlands, and the United States. The sites represented different social and political health systems globally to ascertain the issues that transcend national boundaries in the internationally understood consistent context of PICU. In each hospital, we identified a gatekeeper, who was a clinician (physician or a nurse) working in PICU. This person was not involved in the study design. They invited participants to the study and the core research team subsequently contacted the participants with a formal invitation to interview. Snowball sampling recruited further participants either within the same institution or from other institutions in that country (Hensen et al., 2021). This process meant that all participants self-selected to actively engage with and contribute to the project.

#### Procedures

We interviewed 40 physicians. Due to the COVID-19 pandemic, we interviewed participants remotely using the video conferencing platform Zoom. The interviews were unstructured, and lasted between half an hour and an hour. After a few initial icebreaker questions to establish job role and years of experience, the interviewer asked each participant to talk about a case that they felt was particularly important to them, that involved the initiation or discontinuation of life-sustaining technology. The participants led the conversation, and researchers listened and followed cues as they emerged. Researchers prompted for specific information if necessary, but only if these issues could be naturally included in the conversation. Any questions asked by the researchers were deliberately open-ended and probing in terms of asking for more elaboration or more detail. We kept all data confidential; we transcribed original interview audio recordings, and subsequently anonymized the transcripts using identification codes. We then destroyed the original recordings. We imported the transcripts into NVIVO for analysis.

### Analysis

We used the framework devised by Braun and Clarke for thematic analysis (2006) to analyze the interview data. In this framework, initial open coding followed by detailed categorization facilitated the identification of themes. In each one of these stages we undertook analysis, guided by the analytical framework proposed by Van Manen (1997, 2014a) of tuning to a phenomenon; investigating experience as we live it, not as it is conceptualized; describing through writing and rewriting; and reflecting on essential themes.

## Results

### Demographics

The physicians we interviewed all worked in pediatric or neonatal intensive care. All had at least 5 years’ experience in this area of medicine, with 14 having more than 20 years’ experience. Table 1 supplementary file provides more details about the participants.

### Themes

**Figure 1.**
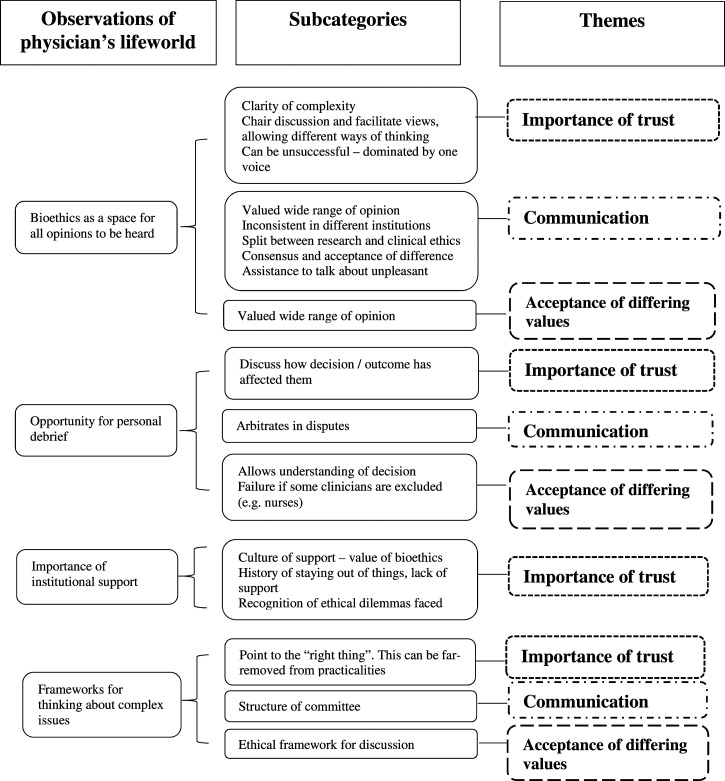
Categories and codes leading to theme development.

The meanings given to the experience of physicians who consult with bioethics services of different structures and using different nomenclature when problem-solving reflected aspects of trust, facilitating communication to aid problem-solving, and acceptance of differing values. These three themes emerged from four types of interactions between physicians and bioethics services at the time when a child requires LTV to sustain life identified as part of the physicians’ lifeworld. These are the use of the bioethics committee as a place where all opinions can be heard and discussed, the opportunity for the bioethics services to provide personal debrief after challenging decisions need to be made; the importance of institutional support for an independent bioethics service; and the frameworks for thinking about complex issues that the bioethics services provide. Figure 1 provides more detail about how the themes developed from the lived experiences of the physicians interviewed.

### The Importance of Trust

The physicians had a strong need for trust in order to feel supported by bioethics, which encompasses the need for confidence that the services would be available when needed and a focus on clinical ethical support; as well as trust in an unbiased service, independent from any organizational influences. The physicians also needed to trust that the bioethics service was not only respectful of the gravity of the decisions they must make, but aware of the pragmatic applications of the ethical dilemmas and discussions in the everyday life of the children, parents, and clinicians.

The physicians expressed appreciation of a bioethics service that was easily accessible and perceived to be useful. The physicians interpreted a service that is hard to access or not supported by the institution as a lack of corporate trust in the benefit of bioethics support, which seems to translate to a feeling of being unsupported or a lack of appreciation of the gravity of the physicians’ dilemmas on the part of the institution:

“we are pushing the limits if you can go home on it [LTV] and these palliative cases and there’s definitely cases you are like I don’t like, I don’t think it’s a good idea, that’s really, you know you are really pushing the ethics limits. I think that our hospital, our personnel administration here they don’t support the physicians when we are saying no” (Intensivist, United States).

The perception that the hospital administration “don’t support” reduces trust in support from the bioethics committee. For example, one intensivist noted that although there remained a relationship with the ethics committee “honestly as a group we don’t really feel, I don’t really feel they add, they add to the conversation” (Intensivist, Australia). In contrast, another participant explained how much easier accessing bioethics services is in their current hospital, compared to a previous working environment where the establishment of a bioethics service remained under discussion rather than being actively developed “I’m not sure if it’s still off the ground, I think people are still discussing it rather than anything else” (Neurologist, Ireland).

Another aspect of trust that we identified concerned a need to feel confident that the bioethics services understood the nuances and the challenges of providing medical care when working at the limits of what is possible in medicine. One participant described how the presence of a mixture of people on a committee helped to address this, increasing the likelihood of the bioethics service to see beyond the “black and white” of any issue. Institutional backing of the bioethics service also affected the physicians’ trust in bioethics in terms of its ability to be supportive. One physician described a case where the physicians’ decision not to support the initiation of technology to sustain life was opposed to the values of the institution:

“our current administration really strongly feel that we shouldn’t deny children of different developmental stages, of different, I don’t know, delayed development what have you … But you know our current administration they try and stay out of things, which I think has the unfortunate consequence of the doctors feeling unsupported and when families like really want something” (Intensivist, United States).

Without the perception of the independent integrity of its members, the physicians were unlikely to perceive the bioethics service as useful in terms of helping with decision-making. One physician expressed how a disengaged bioethics committee dismissed any appreciation of the gravity of decisions in question:

“At least in this country it feels like bioethics is more...more helpful to get a stamp of approval on perhaps a controversial decision” (Respiratory consultant, United States).

A dissonance in the expectation of what could be achieved and the outcome of a consultation seemed to impact negatively on the physicians’ trust and use of the service “we find them not to be particularly helpful … we feel they support neither the family nor the medical team so we just don’t use them anymore” (Intensivist, United States).

The embedding of the decision into the reality of the situation is fundamentally important. If the bioethics team seems to be out of touch or not understanding of the context of any issue, then the physicians actively reject its support, and it is even felt that the bioethics service can even make a situation worse:

“So there might be what appears in the abstract a very ethically sound position that one should adopt in relation to a particular issue. But actually operating it in the real world is unrealistic or could even be cruel you know. I don’t know, say you have a 13 years old or 10 years old who is completely conscious but dependent on life supports and it’s become apparent that their care is now futile. Sort of stuck with a breathing tube and on a ventilator and say they’re getting support for other organs, their heart, their kidneys. An ethics team might say well you have to tell the child that they’re going to die because that’s the right thing to do and they need to know about that” (Intensivist, Ireland).

The physicians’ comments reflect how bioethics’ services that perform a variety of tasks, rather than maintain focus on clinical issues, reduce their trust in its ability to support them. In the views of one physician, clinical ethics, as a form of support for decision-making needs to be focused, and “separate to the ethics committee around getting ethics for your audit, you know which is totally different, different ballgame” (Pediatric specialist consultant, Ireland). The physicians perceived such an apparent lack of focus of the bioethics service as negatively compromising effective discussion. One consultant described how the committee was “more based around research so I don’t think it’s ever been exercised, at least in the ten years or nine years I’ve been here” (Respiratory consultant, Ireland).

### Facilitating Communication to Support Problem-Solving

The physicians in our study regarded the act of communicating safely and effectively as an important function that contributes to how they experience and understand support from bioethics in terms of decision-making and their own well-being. Where bioethics services facilitate communication is by means of providing a specific “ethics space,” and “bringing together people from different parts of the hospital who have a different set of experiences to help sort through” (Complex care consultant, Australia). The framework of bioethical discussion, although not always satisfactory, allows physicians to clarify challenging or conflicting elements of a decision, and helps to resolve any disputes. The perception of bioethics as a decision-maker in itself, however, is less consistently supported.

Physicians felt that bioethics provides “a venue to sort of express their opinions, express their feelings. And really yeah to get that out in terms of any miscommunication” (Intensivist, United States). Some physicians expressed the value in having an expert in bioethics to clarify the main issues needed “to help lay out the pros and cons and help the discussions” (Pediatric specialist consultant, Ireland). Overall, the physicians expressed more respect for the bioethics committee that is representative of many different views, and saw it as supportive if it reflects: “a sort of broad base in terms of including community members and you know clergy and providers from all disciplines” (Intensivist, United States). However, this diversity of opinion is only useful if it is inclusive of all involved in a particular case. A voice that is missing, for example, the exclusion of nurses from a bioethics discussion, is not perceived as ideal, as is the presence of a dominant voice that excludes others from contributing:

“I mean the nice thing about it I suppose is that it tends to include, you know a broader scope of people. But I mean you know for example in [country name] at one point it was just a couple of very unhelpful people on it, so you never got a helpful opinion. And then it just kind of, it just becomes a bit of a roadblock and it’s a pain”. (Intensivist, Australia).

Another potential obstacle to the perception of support given by a larger, diverse committee is when one voice is particularly dominant. If one clinician is unwilling to hear the voices of others, and “they see themselves as the sole person responsible for this child and this family … the others’ input is either not valued or just not relevant” (Intensivist, United States).

Assistance in resolving disputes, or achieving a consensus in treatment decisions, is an important aspect of effective communication involving or facilitating the bioethics services. In many cases, a child presents with a number of comorbidities that contribute to, or complicate, the need for long-term technology initiation. As a means of gaining clarity over a complicated situation, many of the physicians appreciated the input of bioethical thinking and the opportunity for discussion as a facilitating force, even though the physicians make the decisions: “I feel that they’re facilitators often more than giving strict ethical framework.” (Intensivist, Australia). In cases where the discussion centers around the withdrawal of care from a child, even if the child will survive for a short time, the support of bioethics consideration is particularly valued in terms of expanding the topics of communication:

“ICU providers will … get an ethics consult to see if the ethics of, the ethics of not providing life-sustaining technology for a child that will survive. As compared to the debilitated kid that won’t survive and providers won’t offer life-sustaining technologies” (Intensivist, United States)*.*

Some physicians value this open discussion particularly when there are differing professional opinions, for example, in cases where technology dependence is not advised, or it is felt that it is in the child’s best interest to withdraw life-sustaining technological support:

“And we as pediatric intensivists or majority wanted to withdraw the treatment and the neonatologist and one or two of the pediatric intensivists, they wanted to continue it and it was the first time we had a situation within our team and then this ethics committee really helped in getting a better alignment within the team” (Intensivist, Netherlands).

The interviewees also perceived bioethics services as helpful in that they are able to reassure parents when making difficult decisions. The physicians reported that parents as well as health professionals felt reassured that a multi-faceted discussion had taken place, which helps those involved to feel confident that all options have been considered, and that everything has been done that was possible without causing harm to the child.

“there are based from the legal department and also colleagues from the adult ICU and then we consult them and if there is then still, usually in about more than 95% we have consensus within the team and the parents completely understand our decision that we don’t sustain the treatment and we withdraw the therapy” (Intensivist, Netherlands)*.*

Most of the participants expressed how being able to express concerns and opinions among trusted peers is very supportive. This opportunity is individually beneficial, for example, one physician describes his experience of the ethics committee, as “colleagues there who I might discuss things with. And that can be a discussion in a personal way because oh I did this, I’ve had this conversation with parents, it affected me in this or that way” (Physician, Netherlands). Others find support in a less formal setting than in the full committee, but still involving the bioethics service. For example, if those “who are taking care of the child feels a moral problem, we can discuss it and that’s less formal” (Intensivist, Netherlands). Communication of this type seems fundamental in terms of the function of a bioethics service in allowing personal debrief for the physicians from the challenges faced.

If a physician understands the bioethics committee’s communicative role as a primary decision-maker, this negatively influences their perception of its support. A common perception among our participants is that the bioethics service is important in helping to consider the decision, but is not valued as a decision-maker itself:

“I have never yet found an ethics consult to really help very much. Because I think a lot of it is decision by committee. And committees don’t take a stand. They try to give a balanced view of things. Well maybe I don’t come in with a balanced view but like you know, so I’ll be honest, I don’t think we’ve ever had a time when the goals of the patient and the goals of the family have been so far apart that we [physicians and family] can’t figure it out” (Intensivist, United States).

The perception of a “decision by committee” reflects for the physicians a sense of being disengaged from the reality of a situation, or that any resolution is so diluted it does not benefit anyone:

“eventually they got to an end, I wouldn’t say a solution, but they got to an end. And then a couple just kept going until the kid just coded and died. It like sucked” (Intensivist, United States).

### Acceptance of Diverse Values

Many physicians felt that support from bioethics was important in the acceptance of the diverse values of all participants in a discussion around a bioethical issue. The bioethics meeting allows space to discuss distressing issues, address fears of prolonging suffering or doing harm, accepting decisions that may seem suboptimal but tolerable, and helping to move on. This theme is dependent largely on the themes of trust and communication. For example, the bioethics service provides a space to talk about issues that are deeply unpleasant and distressing, for example, when the choices are between two suboptimal options “do we think that it’s fair to the child, is it medically, is it useless what we are doing, are we prolonging suffering of the child?” (Intensivist, Australia). The bioethics consultation in this sense is particularly important in cases where there is dispute, such as if the parents are unable to accept that their choice is causing suffering to the child, or that the physicians feel that this suffering is enough to warrant not giving the technology. The physicians valued the bioethics service as it provides an opportunity to consider all aspects of formulating a treatment plan. The perceived support from bioethics services in helping physicians accept what they judge to be apparently suboptimal situations, particularly if they are not aligned to the personal values, were valued highly by many of the physicians interviewed:

“on the one hand I’d say who am I to overrule that and why is my thought about this kind of situation, why would it be better or more correct or whatever. On the other hand it has, yeah you have got your own way of thinking about these things and it feels wrong to do such a thing. So often we would, yeah we would have discussion with the whole team, if we find ourselves in such a situation”*.* (Intensivist, Australia).

An important means of achieving this support is by ensuring that the bioethics discussion is comprehensive. Physicians regarded the bioethics service as valuable in helping those with different opinions to accept a status quo that for them is less than optimal, but nevertheless adequate. Our participants appreciated this means of avoiding distress, or at least coping with distress, and enabling them to continue working effectively in a team:

“So we’ve had discussions where there have been complex decisions at a round table with an ethical consult, with a formal ethics group and we all accept the fact that once a decision is made you move on” (Intensivist, Australia).

## Discussion

This research has identified key elements of the perceptions physicians have of their relationship with bioethics as a supportive or unsupportive service, which seem common across the different countries in this study. Within the research team there was a continuous focus and discussion on the criteria for rigor by Guba and Lincoln (1982). This was extremely valuable in decision-making on every element of the study, particularly when gathering and analyzing data during the unprecedented pandemic.

**Figure 2.**
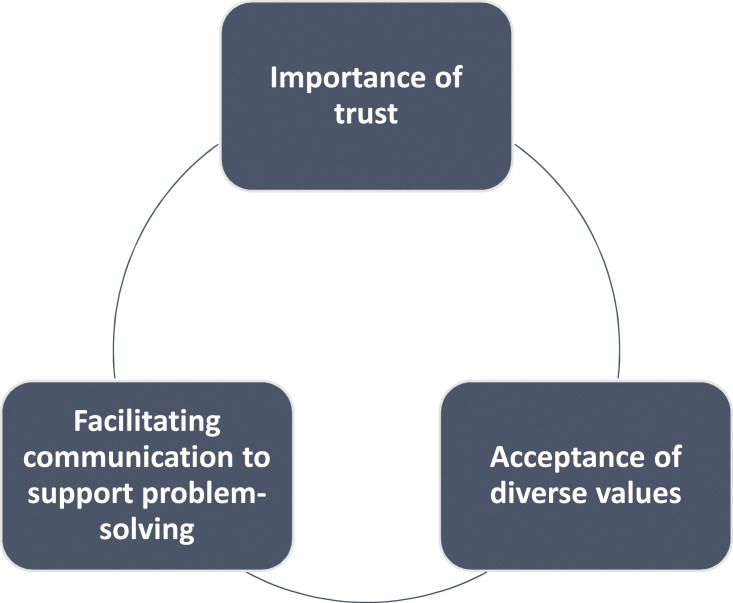
Model for successful functioning of bioethics services to support clinical care.

Managing the human consequences of making such decisions while caring for a profoundly ill child is highly charged and complex. The physicians in our study all understood this experience to be an essential and generally rewarding part of their work, an attitude echoed by Thorne et al. (2018) in an exploration of PICU nurses. The physicians understand and perceive bioethics as support in different ways: as part of their institution and working life, as a framework on which to base discussions, as a focal point and arena for open discussions, and on an individual level as a means of personal support. What makes these interactions satisfactory or unsatisfactory to the physicians are the presence or absence of trust, communication, and acceptance of values. The discussion is structured around these three key themes, reflecting the literature and key findings on the successful functioning of bioethics services to support clinical care, which collectively form a model for successful functioning of bioethics services to support clinical care (Figure 2).

### The Importance of Trust

As with all successful relationships, trust within the involved parties is essential, particularly in cases where a child’s life or death, and the future personal, family, social, and economic well-being is the object of the discussion. The notion of bioethics input and services being in a place of trust seems essential in order for physicians to feel supported in decision-making at this point in a child’s care. Placencia and McCullough (2017) discuss the importance of bioethics services in assisting decision-making in a neonatal context; particularly as the context of such decisions is highly charged and emotional (Cricco-Lizza, 2014). Wocial et al. (2017) stress the value of timely bioethics consultations to lessen conflict and moral distress among physicians and parents, as well as reducing the time spent in making decisions in PICU. Our participants expressed similar views in this research. Wocial et al. (2017) observed that trust in the bioethics team was present when its input was proactive and timely; as opposed to bureaucratic, intimidating and distant from the lived experiences of the physicians. Our research found that when the bioethics services were accessible and appreciative of the nuances of decisions and consequences, the physicians felt supported. Conversely, if they felt that bioethics lacked understanding of the context of critical decisions, the service had little to offer above what the physicians could decide amongst themselves; in a few cases, physicians perceived the bioethics service as actively harmful.

Consistent with the literature, our study found that there are different expectations of the role of bioethics services in clinical deliberations. This mirrors our findings in the literature that bioethics services are nationally and internationally very different in their remit and their membership (Hajibabaee et al., 2016; Kesselheim et al., 2010). Much of the research into bioethics services are from single-center sites, for example, Gold et al. (2011), Jansen et al. (2018), McDougall and Notini (2016). Kon et al. (2016) describe physicians’ criticism of bioethics for not providing an answer, or not having the right answers in challenging situations. A survey by Bucher et al. (2018), found that nearly half of the health professionals in PICU considered hospital bioethics services to be the “ultimate decision maker,” compared to one tenth who felt that this role should be carried out by the physicians, and just over a third who felt that it was the parents who had the final say. In contrast, research by Abdel Razeq (2019) found that although most physicians felt that bioethics involvement was beneficial in end-of-life decision-making, most felt that the bioethics role should be in setting general guidelines and facilitating discussions rather than intervening in decisions about individual cases. These differences in expectation may well contribute to the variation in felt support from bioethics expressed by physicians in this study. Some felt they should make decisions without any bioethical assistance, and only use bioethics services as a last resort, an attitude echoed by Nguyen and Ho (2013). This situation is in contrast to what we know about the remit of clinical bioethics services by clinicians. There is a gap in expectation or understanding of the role and capacity between these two professions, which demonstrates an urgent need for education.

Another important facet of trust is that bioethics relies upon professional trust between all involved parties (Stevens et al., 2011; Thorne et al., 2018). Bioethics discussions involve many different individuals, all of whom can review the same evidence and come to different conclusions. The art of consensus relies upon respecting the views and productive discussions to agree a way forward (Wilkinson & Truog, 2013).

Where there is trust that the bioethics service conduct discussions with good knowledge of the context and nuances of each decision and its ramifications, the physicians were more likely to engage. In our study, the physicians who had had good experiences, and resulting trust in the benefits of bioethics discussions, felt more supported than those who had negative experiences, or who had felt let down. Reiter-Theil (2012) concluded that ethics must acknowledge empirical research, and that there is always a gap between ethical norms and the human practice. Although the ethical norm must take precedence, an element of pragmatism is essential. One way of acknowledging this need for pragmatism is the use of what has been termed the parental zone of discretion (Lantos, 2018). Where a treatment cannot be ethically determined as providing clear benefit, or clear harm, then a pragmatic decision must be made either in terms of the child’s right to treatment, or by following the choices of the parents (Kon et al., 2016). However, in practice, the optimal choice can be difficult to determine, and trust in all those involved is essential. There are no exact delineations of the parental zone of discretion; individual circumstances, the condition of the child as well as social, political, cultural, and other norms of behavior influence the decisions (Lantos, 2018). An element of trust in the integrity and professionalism of all partners is essential for this aspect of bioethical and clinical decision to function (Stevens et al., 2011; Thorne et al., 2018).

We found that the physicians who felt more supported by bioethics were more likely to use the services to help them make shared decisions, or to communicate with parents in terms of what would be possible for their child. Lantos (2018) argues that conducting long deliberate sessions of discussion to find a solution to a child’s care is not necessarily part of the physician’s skill set. However, genuinely shared decision-making necessary for the initiation of long-term technology, or its withdrawal, can be very challenging. This situation is where trust from bioethics services is vital to ensure the identification and amelioration of implicit bias and irrational thought patterns or beliefs in group-discussions, which is particularly important when there is no clear direction of treatment for an individual child (Lantos, 2018).

### Facilitating Communication to Support Problem-Solving

Being able to discuss sensitive and difficult issues within a group, not only depends upon elements of trust, but also in being able to communicate effectively. Our research found that having diversity of opinion and of professions in bioethics committees or meetings were valued by physicians, which was perceived as supportive in helping resolve differences and come to a treatment decision about a complex issue. Roscigno and Swanson (2011) demonstrate the importance of broad communication, and Morrow (2015) underlines the benefit of seeking advice from experienced clinical ethics committee in the context of withdrawing technology at the end of life. Over time, bioethics committees have attempted to evolve to meet changing needs, for example, moving from domination by physicians to becoming more interdisciplinary in order to achieve better communication. The nature of the current discussions have consequences far outside the purely medical but may influence future issues for many years (Henderson et al., 2018). Our respondents felt that if representation was not comprehensive, for example, if nurses were included or if one individual dominated the discussion, which undermined the value of an interdisciplinary bioethics committee. One important aspect of communication valued by our participants was a recognition of the need for the bioethics service to manage a discussion and help clarify the goals of care for each individual child in order to facilitate a conclusion. This function is reflected in the literature, which shows how bioethics is regarded by many clinicians as a useful means of working through a problem (Johnson et al., 2015; McDougall & Notini, 2016; Rallison & Raffin-Bouchal, 2013). A challenge to achieving this support occurs if the ethics committee is not easily accessible. As Linney et al. (2019) suggest, it can take time to access bioethics support, and time to resolve difficult ethical issues. Bioethics services that are more agile, whether they contain large committees or consist of smaller, more ad hoc ethical discussions, seem to be more valued where they are available (Leland et al., 2020). In wider discussions about the ethical climate in PICU, a core contributor to a positive ethical climate was the facilitation of equal involvement of interdisciplinary professionals in ethical discussions about the care of children with technology dependence in PICU (Moynihan et al., 2021).

Participants in this study expressed that they often only consult bioethics services if there is a situation that seems unresolvable, or there is a concern about a dispute with the child’s family. Johnson et al. (2015) found that family constraints on physicians ability to treat, or if the family is insistent on treatment considered unnecessary, is a major cause of moral distress among clinicians. Although the research on bioethics services suggests that a means of avoiding dispute is to engage earlier in the process, in reality early engagement does not always happen (Weise et al., 2017). Preisz (2019) points out that physicians’ training in decision-making is generally oriented towards agile thinking that results in confident and immediate decisions. The slow, deliberative nature of shared decision-making around the initiation of life-sustaining technology is very different and can be very difficult to achieve. The input of the bioethics service can be invaluable in guiding the communication through the use of an ethical framework; and as a result, helping satisfy all parties that the best path is being taken (Lantos, 2018).

### Acceptance of Diverse Values

Professionally it is difficult, if not damaging, to continue working in an environment where you do not agree with a decision. The literature surrounding moral distress discusses this at length (Hamric, 2014; Johnson et al., 2015). The physicians in our study generally expressed the need to accept decisions and move on in order to work effectively within their teams, and valued the contribution of bioethics services in order to help them understand the reasoning behind a decision (Kirsch & Munson, 2018). This process does not change the reality of the situation but allows the individual clinician to understand the rationale. There is no requirement to agree, rather there is to understand. Without such support, living through these dilemmas can become mentally harmful.

To a great extent it is the trust in bioethics services to help with decision-making, and effective communication that contributes to the ability to accept decisions that are not necessarily aligned with individual views and values. In addition, physicians more readily accept input from bioethics when there is also consideration for the wider cultural context of any decision. Brick et al. (2020) found that there were considerable differences between the views of bioethics, and those of the public. There also remains an important influence of physician’ and parental values in any decision that is made (Roscigno et al., 2012). Johnson et al., (2015) found that even when shared decision-making is sought, physicians retained a “protective paternalism” (p. 13) towards their patients, that the care goals align with “the clinicians’ personal sense of what would be in the child’s best interest” (p. 13). The researchers proposed that this perception might influence physician reticence to seek bioethics input to support their decision-making. In our research, we found that if the bioethics services were not valued, or were viewed as a threat to professional decision-making, then acceptance was harder to gain. Hamric’s research (2014) on moral distress also showed that reasons why bioethics were not consulted included fear that they would take over, or the implication that a bioethics consultation would imply that the physician team was inadequate or unable to do a good job. This misconception was also implied by some of our participants, who felt that bioethics committees who were reluctant to make a decision were not worth consulting; and others who felt that the bioethics have no place in making a decision. The confusion in expectation of what the bioethics services should be for is a reflection of the differences in bioethics services across countries and institutions. This situation interfered with the ability of physicians to accept situations, and reduced felt perceptions of bioethics as a source of support.

### Limitations

We were unable to interview participants face-to-face because of the COVID-19 pandemic in 2020. Instead, we interviewed remotely using Zoom video conferencing. This restriction potentially limited opportunity for interpretation of the meanings of the conversations in terms of non-verbal engagement, but we took additional steps before each interview to build a relationship with the participant, such as a series of short emails to introduce the researcher, the project, and the web link for interview. These measures helped to ensure that the interviewee felt as relaxed as possible before and during the interview process (De Chesnay, 2014, pp. 20–21 & pp. 53–56; Howitt, 2019, pp. 65–66). In addition, conceptualizing a bioethics service and its functions was difficult because of the wide variation across settings and countries. Nevertheless, this research aimed to understand the lived experiences of the physicians’ perspectives on the bioethics services they encounter.

## Conclusion

This exploration has provided a picture of the lived experiences of physicians of the functions of bioethics at the point of care when a child is likely to need technology dependence to survive. The success of these functions depends upon: the foundations of trust and integrity, facilitating communication and acceptance of situations that are challenging and, largely, beyond further control of the physicians. This conceptual triad of dependencies offers a model to guide future research in this area. The physicians expressed the need to share the burden of ethical decision-making and implied that doing so is essential in order to continue caring for children who are at the edge of life. Bioethics services have the potential to help physicians gain clarity and objectivity over complex situations, provide advice and support in their considerations. In some institutions, it is very successful in performing these functions. However, where the bioethics services are disengaged from the work of the clinicians, either because of a lack of institutional commitment, a lack of independence, or lack of knowledge, the perception of support from bioethics by physicians is negatively affected. This negativity can increase beyond feelings of being unsupported, to an avoidance of bioethical help, despite evidence to suggest its potential utility, and moving towards a perception that the service is out-of-touch or potentially actively harmful.

The perceptions of bioethics from the clinicians interviewed showed that establishing clarity in the purpose and remit of bioethics’ services in their contribution to medical deliberations is vitally important. If the clinician expects all discussions with bioethics to conclude with a final decision, it can be frustrating when it does not happen. On the other hand, if the physician regards bioethics as an outside decision-making agency, disconnected from the reality of PICU, the service may be resented. Indeed, in this situation it is seen as a sign of failure if a physician has to “resort to ethics” if they have been unable to conclude a discussion themselves. In this type of scenario, rather than a source of support, bioethics becomes a source of threat.

The bioethics services themselves need to tread a fine line to make working at this point in care delivery easier. Successful support depends upon understanding of each participant’s values and aims for any consultation. In addition, pragmatic awareness of the wider cultural environment seems to improve perception of the support provided by bioethics input into decisions. Future research should explore the nature of communication between physicians and bioethics services and the effect of different structures of bioethics services; the perspectives of the members of bioethics services; the perspective of parents; and the perspectives of other health care professionals, and hospital boards in terms of the changing environment of technological medicine in PICU. Decision-making for children required life-sustaining technology is becoming increasingly challenging and this research would support further development of trust, communication and acceptance of values across the PICUs and bioethics services.

## Supplemental Material

sj-pdf-1-qhr-10.1177_10497323221083744 – Supplemental Material for The Meaning Given to Bioethics as a Source of Support by Physicians Who Care for Children Who Require Long-Term Ventilation Click here for additional data file. Supplemental Material, sj-pdf-1-qhr-10.1177_10497323221083744 for The Meaning Given to Bioethics as a Source of Support by Physicians Who Care for Children Who Require Long-Term Ventilation by Denise Alexander, Mary B. Quirke, Carmel Doyle, Katie Hill, Kate Masterson and Maria Brenner in Qualitative Health Research

## References

[bibr1-10497323221083744] Abdel Razeq N. M. (2019). Physicians’ standpoints on end-of-life decisions at the neonatal intensive care units in Jordan. Journal of Child Health Care, 23(4), 579–595. 10.1177/1367493518814926. 30606043

[bibr2-10497323221083744] Alexander D. Eustace-Cook J. Brenner M. (2021b). Approaches to the initiation of life-sustaining technology in children: a scoping review of changes over time. Journal of Child Health Care, 25(4), 509–522. 10.1177/1367493520961884. 32966106

[bibr3-10497323221083744] Alexander D. Quirke M. B. Berry J. Eustace-Cook J. Leroy P. Masterson K. Healy M. Brenner M. (2021a). Initiating technology dependence to sustain a child’s life: a systematic review of reasons. Journal of Medical Ethics. Published Online First. 10.1136/medethics-2020-107099. PMC9726963 34282042

[bibr4-10497323221083744] Amin R. Sayal P. Syed F. Chaves A. Moraes T. J. MacLusky I. (2014). Pediatric long-term home mechanical ventilation: Twenty years of follow-up from one Canadian center. Pediatric Pulmonolology, 49(8), 816–824. 10.1002/ppul.22868. 24000198

[bibr5-10497323221083744] Beauchamp T. L. Childress J. F. (1989). Principles of biomedical ethics (3rd ed.). Oxford University Press.

[bibr6-10497323221083744] Braun V. Clarke V. (2006). Using thematic analysis in psychology. Qualitative Research in Psychology, 3(2), 77–101. 10.1191/1478088706qp063oa.

[bibr7-10497323221083744] Brick C. Kahane G. Wilkinson D. Caviola L. Savulescu J. (2020). Worth living or worth dying? The views of the general public about allowing disabled children to die. Journal of Medical Ethics, 46(1), 7–15. 10.1136/medethics-2019-105639. 31615879 PMC6984061

[bibr8-10497323221083744] Bucher H. U. Klein S. D. Hendriks M. J. Baumann-Hölzle R. Berger T. M. Streuli J. C. Fauchère J-C. (2018). Decision-making at the limit of viability: Differing perceptions and opinions between neonatal physicians and nurses. BMC Pediatrics, 18(1), 81. 10.1186/s12887-018-1040-z. 29471821 PMC5822553

[bibr9-10497323221083744] Carel H. (2011). Phenomenology and its application in medicine. Theoretical Medicine and Bioethics, 32(1), 33–46. 10.1007/s11017-010-9161-x. 21103940

[bibr10-10497323221083744] Carnevale F. A. (2005). Ethical care of the critically ill child: A conception of a ‘thick’ bioethics. Nursing Ethics, 12(3), 239–252. 10.1191/0969733005ne786oa. 15921341

[bibr11-10497323221083744] Cohen E. Kuo D. Z. Agrawal R. Berry J. G. Bhagat S. K. M. Simon T. D. Srivastava R. (2011). Children with medical complexity: An emerging population for clinical and research initiatives. Pediatrics, 127(3), 529–538. 10.1542/peds.2010-0910. 21339266 PMC3387912

[bibr12-10497323221083744] Cottle E. Jansen M. Irving H. Mathews B. (2017). Paediatric clinical ethics in Australia and New Zealand: A survey. BMJ Paediatrics Open, 1(1), Article e000156. 10.1136/bmjpo-2017-000156. 29637160 PMC5862174

[bibr13-10497323221083744] Cricco-Lizza R. (2014). The need to nurse the nurse: Emotional labor in neonatal intensive care. Qualitative Health Research, 24(5), 615–628. 10.1177/1049732314528810. 24675967

[bibr14-10497323221083744] De Chesnay M. (2014). Nursing research using phenomenology: Qualitative designs and methods in nursing. Springer Publishing Company. https://search-ebscohost-com.elib.tcd.ie/login.aspx?direct=true&db=nlebk&AN=910078&site=ehost-live.

[bibr15-10497323221083744] Fontana M. S. Farrell C. Gauvin F. Lacroix J. Janvier A. (2013). Modes of death in pediatrics: Differences in the ethical approach in neonatal and pediatric patients. The Journal of Pediatrics, 162(6), 1107–1111. 10.1016/j.jpeds.2012.12.008. 23312685

[bibr16-10497323221083744] Gillon R. (1994). Medical ethics: Four principles plus attention to scope. BMJ: British Medical Journal, 309(6948), 184-184. 10.1136/bmj.309.6948.184. 8044100 PMC2540719

[bibr17-10497323221083744] Gold H. Hall G. Gillam L. (2011). Role and function of a paediatric clinical ethics service: Experiences at the Royal Children’s Hospital, Melbourne. Journal of Paediatrics and Child Health, 47(9), 632–636. 10.1111/j.1440-1754.2011.02171.x. 21951448

[bibr18-10497323221083744] Guba E. Lincoln Y. S. (1982). Epistemological and methodological bases of naturalistic inquiry. Educational Communication and Technology, 30(4), 233–252. https://www.jstor.org/stable/30219846.

[bibr19-10497323221083744] Hajibabaee F. Joolaee S. Cheraghi M. A. Salari P. Rodney P. (2016). Hospital/clinical ethics committees’ notion: An overview. Journal of Medical Ethics and History of Medicine, 9(7), 17. https://www-ncbi-nlm-nih-gov.elib.tcd.ie/pmc/articles/PMC5432947/pdf/JMEHM-9-17.pdf. 28523118 PMC5432947

[bibr20-10497323221083744] Hamric A. B. (2014). A case study of moral distress. Journal of Hospice & Palliative Nursing, 16(8), 457–463. 10.1097/NJH.0000000000000104.

[bibr21-10497323221083744] Henderson C. M. Wilfond B .S. Boss R. D. (2018). Bringing social context into the conversation about pediatric long-term ventilation. Hospital Pediatrics, 8(2), 102–108. 10.1542/hpeds.2016-0168. 29326228

[bibr22-10497323221083744] Hensen B. Mackworth-Young C. Simwinga M. Abdelmagid N. Banda J. Mavodza C. Doyle A. M. Bonell C. Weiss H. A. (2021). Remote data collection for public health research in a COVID-19 era: Ethical implications, challenges and opportunities. Health Policy and Planning, 36(3), 360–368. 10.1093/heapol/czaa158. 33881138 PMC7928874

[bibr23-10497323221083744] Howitt D. (2019). Introduction to qualitative research methods in psychology. Pearson Education, Limited.

[bibr24-10497323221083744] Jansen M. A. Schlapbach L. J. Irving H. (2018). Evaluation of a paediatric clinical ethics service. Journal of Paediatrics and Child Health, 54(11), 1199–1205. 10.1111/jpc.13933. 29746009

[bibr25-10497323221083744] Johnson L.-M. Church C. L. Metzger M. Baker J. N. (2015). Ethics consultation in pediatrics: Long-term experience from a pediatric oncology center. The American Journal of Bioethics, 15(5), 3–17. 10.1080/15265161.2015.1021965. PMC4685463 25970382

[bibr26-10497323221083744] Kesselheim J. C. Johnson J. Joffe S. (2010). Ethics consultation in children’s hospitals: Results from a survey of pediatric clinical ethicists. Pediatrics, 125(4), 742–746. 10.1542/peds.2009-1813. 20194289

[bibr27-10497323221083744] Kirsch R. Munson D. (2018). Ethical and end of life considerations for neonates requiring ECMO support. Seminars in Perinatology, 42(2), 129–137. 10.1053/j.semperi.2017.12.009. 29331209

[bibr28-10497323221083744] Kon A. A. Patel A. Leuthner S. Lantos J. (2016). Parental refusal of surgery in an infant with tricuspid atresia. Pediatrics, 138(5), e20161730. 10.1542/peds.2016-1730. 27940784

[bibr29-10497323221083744] Lantos J. D. (2018). Ethical problems in decision making in the neonatal ICU. New England Journal of Medicine, 379(19), 1851–1860. 10.1056/NEJMra1801063. 30403936

[bibr30-10497323221083744] Leland B. D. Wocial L. D. Drury K. Rowan C. M. Helft P. R. Torke A. M. (2020). Development and retrospective review of a pediatric ethics consultation service at a large academic center. HEC Forum, 32(3), 269–281. 10.1007/s10730-020-09397-6. 32180057

[bibr31-10497323221083744] Linney M. Hain R. D. W. Wilkinson D. Fortune P-M. Barclay S. Larcher V. Fitzgerald J. Arkell E. (2019). Achieving consensus advice for paediatricians and other health professionals: On prevention, recognition and management of conflict in paediatric practice. Archives of Disease in Childhood, 104(5), 413–416. 10.1136/archdischild-2018-316485. 31000533 PMC6557224

[bibr32-10497323221083744] McDougall R. J. Notini L. (2016). What kinds of cases do paediatricians refer to clinical ethics? Insights from 184 case referrals at an Australian paediatric hospital. Journal of Medical Ethics, 42(9), 586–591. 10.1136/medethics-2015-103025. 27317508

[bibr33-10497323221083744] Morrison W. Womer J. Nathanson P. Kersun L. Hester D. M. Walsh C. Feudtner C. (2015). Pediatricians’ experience with clinical ethics consultation: A National Survey. Journal of Pediatrics, 167(4), 919–924. 10.1016/j.jpeds.2015.06.047. 26210945

[bibr34-10497323221083744] Morrow B. M. (2015). End-of-life care in the pediatric intensive care units: Challenges and ethical principles. Indian Journal of Critical Care Medicine, 19(3), 133–135. 10.4103/0972-5229.152749. 25810605 PMC4366908

[bibr35-10497323221083744] Moynihan K. M. Taylor L. Crowe L. Balnaves M-C. Irving H. Ozonoff A. Truog R. D. Jansen M. (2021). Ethical climate in contemporary paediatric intensive care. Journal of Medical Ethics. 10.1136/medethics-2020-106818. 33431646

[bibr36-10497323221083744] Nguyen T. A. P. Ho L. Y. (2013). Review on neonatal end-of-life decision making: medical authority or parental autonomy? Proceedings of Singapore Healthcare, 22(2), 140–145. 10.1177/201010581302200210.

[bibr37-10497323221083744] Norlyk A. Harder I. (2010). What makes a phenomenological study phenomenological? An analysis of peer-reviewed empirical nursing studies. Qualitative Health Research, 20(3), 420–431. 10.1177/1049732309357435. 20068190

[bibr38-10497323221083744] Novotny W. E. Perkin R. M. Mukherjee D. Lantos J. D. (2014). Mechanical ventilation for a child with quadriplegia. Pediatrics, 134(3), 593–597. 10.1542/peds.2013-4161. 25136041

[bibr39-10497323221083744] Placencia F. X. McCullough L. B. (2017). The history of ethical decision making in neonatal intensive care. Journal of Intensive Care Medicine, 26(6), 368–384. 10.1177/0885066610393315. 21606057

[bibr40-10497323221083744] Preisz A. (2019). Fast and slow thinking and the problem of conflating clinical reasoning and ethical deliberation in acute decision-making. Journal of Paediatrics and Child Health, 55(6), 621–624. 10.1111/jpc.14447. 30932284

[bibr60-10497323221083744] Rallison L. B. Raffin-Bouchal S. (2013) (In press). Living in the in-Between: Families caring for a child with a progressive neurodegenerative illness. Qualitative Health Research, 23(2), 194–206. 10.1177/1049732312467232. 23175537

[bibr41-10497323221083744] Reiter-Theil S. (2012). What does empirical research contribute to medical ethics? Cambridge Quarterly of Healthcare Ethics, 21(4), 425–435. 10.1017/S0963180112000205. 22828037

[bibr42-10497323221083744] Rodriguez A. Smith J. (2018). Phenomenology as a healthcare research method. Evidence Based Nursing, 21(4), 96–98. 10.1136/eb-2018-102990. 30201830

[bibr43-10497323221083744] Roscigno C. I. Savage T. A. Kavanaugh K. Moro T. T. Kilpatrick S. J. Strassner H. T. Grobman W. A. Kimura R. E. (2012). Divergent views of hope influencing communications between parents and hospital providers. Qualitative Health Research, 22(9), 1232–1246. 10.1177/1049732312449210. 22745363 PMC3572714

[bibr44-10497323221083744] Roscigno C. I. Swanson K. M. (2011). Parents’ experiences following children’s moderate to severe traumatic brain injury: A clash of cultures. Qualitative Health Research, 21(10), 1413–1426. 10.1177/1049732311410988. 21613654 PMC3444164

[bibr45-10497323221083744] Shapiro M. C. Donohue P. K. Kudchadkar S. R. Hutton N. Boss R. D. (2017). Professional responsibility, consensus, and conflict: A survey of physician decisions for the chronically critically ill in neonatal and pediatric intensive care units. Pediatric Critical Care Medicine, 18(9), e415–e422. 10.1097/PCC.0000000000001247. 28658198

[bibr46-10497323221083744] Stevens B. Riahi S. Cardoso R. Ballantyne M. Yamada J. Beyene J. Breau L. Camfield C. Finley G. A. Franck L. Gibbins S. Howlett A. McGrath P. J. McKeever P. O'Brien K. Ohlsson A. (2011). The influence of context on pain practices in the NICU: Perceptions of health care professionals. Qualitative Health Research, 21(6), 757–770. 10.1177/1049732311400628. 21357756

[bibr47-10497323221083744] Streuli J. C. Staubli G. Pfändler-Poletti M. Baumann-Hölzle R. Ersch J. (2014). Five-year experience of clinical ethics consultations in a pediatric teaching hospital. European Journal of Pediatrics, 173(5), 629–636. 10.1007/s00431-013-2221-2. 24323344

[bibr48-10497323221083744] Thomas S. M. Ford P. J. Weise K. L. Worley S. Kodish E. (2015). Not just little adults: A review of 102 paediatric ethics consultations. Acta Paediatrica, 104(5), 529–534. 10.1111/apa.12940. 25611088

[bibr49-10497323221083744] Thorne S. Konikoff L. Brown H. Albersheim S. (2018). Navigating the dangerous terrain of moral distress: Understanding response patterns in the NICU. Qualitative Health Research, 28(5), 683–701. 10.1177/10497323177535. 29357751

[bibr50-10497323221083744] Van Manen M. (1997). Researching lived experience: Human science for an action sensitive pedagogy (2nd ed.). The Althouse Press.

[bibr51-10497323221083744] Van Manen M. (2014a). Phenomenology of practice, meaning-giving methods in phenomenological research and writing. Routledge.

[bibr54-10497323221083744] Van Manen M. A. (2014b). On ethical (in)decisions experienced by parents of infants in neonatal intensive care. Qualitative Health Research, 24(2), 279–287. 10.1177/1049732313520081. 24469694

[bibr52-10497323221083744] Van Manen M. (2017a). Phenomenology in its original sense. Qualitative Health Research, 27(6), 810–825. 10.1177/1049732317699381. 28682720

[bibr53-10497323221083744] Van Manen M. (2017b). The ventricular assist device in the life of the child: A phenomenological pediatric study. Qualitative Health Research, 27(6), 792–804. 10.1177/104973231770085. 28682718 PMC5405822

[bibr55-10497323221083744] Wallis C. Paton J. Y. Beaton S. Jardine E. (2011). Children on long-term ventilatory support: 10 years of progress. Archives of Disease in Childhood, 96(11), 998–1002. 10.1136/adc.2010.192864. 21109507

[bibr56-10497323221083744] Weise K. L. Okun A. L. Carter B. S. Christian C. W. (2017). Guidance on forgoing life-sustaining medical treatment. Pediatrics, 140(3), Article e20171905. 10.1542/peds.2017-1905. 28847979

[bibr57-10497323221083744] Weiss S Van Egmond-Fröhlich A Hofer N. Pfleger A. Rath R. Kurz H. Schwarz R. Kurz H. Waibel V. Kenzian H. Kommer E. Wadlegger F. Stelzl W. Keck B. Grigorow I. Kerbl R. Sauseng W. Frischer T. Eber E. Bernert G. (2016). Long-term respiratory support for children and adolescents in Austria: A national survey. Klinische Padiatrie, 228(1), 42–46. 10.1055/s-0035-1565240. 26697738

[bibr58-10497323221083744] Wilkinson D. J. C. Truog R. D. (2013). The luck of the draw: Physician-related variability in end-of-life decision-making in intensive care. Intensive Care Medicine, 39(6), 1128–1132. 10.1007/s00134-013-2871-6. 23435951

[bibr59-10497323221083744] Wocial L. Ackerman V. Leland B. Benneyworth B. Patel V. Tong Y. Nitu M. (2017). Pediatric ethics and communication excellence (PEACE) rounds: Decreasing moral distress and patient length of stay in the PICU. HEC Forum, 29(1), 75–91. 10.1007/s10730-016-9313-0. 27815753

