# Supplementary material for: The Meaning Given to Bioethics as a Source of Support by Physicians Who Care for Children Who Require Long-Term Ventilation
Source: Qual Health Res. 2022 Mar 29;32(6):916–28. doi: 10.1177/10497323221083744 (PMC9189592; doi:10.1177/10497323221083744)
Supplement: sj-pdf-1-qhr-10.1177_10497323221083744 – Supplemental Material for The Meaning Given to Bioethics as a Source of Support by Physicians Who Care for Children Who Require Long-Term Ventilation [file sj-pdf-1-qhr-10.1177_10497323221083744.pdf]

Supplementary File Table 1: Interview participant demographics

Country	Current job title	Overall years of experience	Years in current post
Australia	Specialist Pediatrician	20-24 years	20-24 years
	Specialist Pediatrician	15-19 years	15-19 years
	Cardiac intensivist	Did not say	20 years
	PICU Consultant / intensivist	10-14 years	0-4 years
	PICU Consultant / intensivist	10-14 years	5-9 years
	PICU Consultant / intensivist	>30 years	10-14 years
	PICU Fellow	5-10 years	0-4 years
Ireland	Specialist Pediatrician	25-29 years	20-24 years
	Specialist Pediatrician	15-19 years	5-9 years
	PICU Consultant / intensivist	25-29 years	20-24 years
	PICU Consultant / intensivist	15-19 years	5-9 years
	PICU Consultant / intensivist	10-14 years	10-14 years
	PICU Consultant / intensivist	25-29 years	15-19 years
	PICU Consultant / intensivist	20-24 years	10-14 years
	PICU Consultant / intensivist	5-9 years	0-4 years
	Pediatric Respiratory consultant	10-14 years	5-9 years
	Pediatric Respiratory consultant	20-24 years	5-9 years
	Pediatric Respiratory consultant	15-19 years	5-9 years
	Pediatric Respiratory consultant	25-29 years	15-19 years
Netherlands	Specialist Pediatrician	5-9 years	5-9 years
	Neonatologist	10-14 years	0-4 years
	Neonatologist	10-14 years	0-4 years
	Specialist Pediatrician	15-19 years	15-9 years
	Specialist Pediatrician	10-14 years	10-14 years
	PICU Consultant / intensivist	5-9 years	0-4 years
	PICU Consultant / intensivist	20-24 years	0-4 years
	PICU Consultant / intensivist	5-9 years	0-4 years
	PICU Consultant / intensivist	20-24 years	0-4 years
	PICU Consultant / intensivist	15-19 years	15-19 years
	PICU Fellow	5-9 years	0-4 year
	Pediatric Respiratory consultant	25-29 years	15-19 years
United States	Neonatologist	Did not say	10-14 years
	PICU Consultant / intensivist	Did not say	15-19 years
	PICU Consultant / intensivist	Did not say	5-9 years
	PICU Consultant / intensivist	20-24 years	10-14 years

	PICU Consultant / intensivist	>30 years	>30 years
	PICU Consultant / intensivist	20-24 years	15-19 years
	PICU Consultant / intensivist	5-9 years	0-4 years
	Pediatric Respiratory consultant	Did not say	>30 years
	Pediatric Respiratory consultant	Did not say	10-14 years
